# Nonhomologous Chromosome Interactions in Prophase I: Dynamics of Bizarre Meiotic Contacts in the Alay Mole Vole *Ellobius alaicus* (Mammalia, Rodentia)

**DOI:** 10.3390/genes13122196

**Published:** 2022-11-23

**Authors:** Sergey Matveevsky, Irina Bakloushinskaya, Valentina Tambovtseva, Maret Atsaeva, Tatiana Grishaeva, Aleksey Bogdanov, Oxana Kolomiets

**Affiliations:** 1 Vavilov Institute of General Genetics, Russian Academy of Sciences, 119991 Moscow, Russia; 2 Koltzov Institute of Developmental Biology, Russian Academy of Sciences, 119334 Moscow, Russia; 3 Department of Cell Biology, Morphology and Microbiology, Chechen State University, 364024 Grozny, Russia

**Keywords:** meiotic nonhomologous chromosome contacts, zygotene, diplotene, synaptonemal complex, *Ellobius*

## Abstract

Nonhomologous chromosome interactions take place in both somatic and meiotic cells. Prior to this study, we had discovered special contacts through the SYCP3 (synaptonemal complex protein 3) filament between the short arms of nonhomologous acrocentrics at the pachytene stage in the Alay mole vole, and these contacts demonstrate several patterns from proximity to the complete fusion stage. Here, we investigated the nonhomologous chromosome contacts in meiotic prophase I. It turned out that such contacts do not introduce changes into the classic distribution of DNA double-strand breaks. It is noteworthy that not all meiotic contacts were localized in the H3k9me3-positive heterochromatic environment. Both in the mid zygotene and in the early–mid diplotene, three types of contacts (proximity, touching, and anchoring/tethering) were observed, whereas fusion seems to be characteristic only for pachytene. The number of contacts in the mid pachytene is significantly higher than that in the zygotene, and the distance between centromeres in nonhomologous contacts is also the smallest in mid pachytene for all types of contacts. Thus, this work provides a new insight into the behavior of meiotic contacts during prophase I and points to avenues of further research.

## 1. Introduction

In the first meiotic division, homologous chromosomes undergo many events that eventually lead to interhomolog physical linkages called crossovers, which result in recombination. Homologs are aligned precisely with each other and then synapse via the nucleoprotein synaptonemal complex (SC) and repair programmed DNA double-strand breaks (DSBs) [1]. At this time point, the homologous chromosomes are attached by their telomeric segments to the nuclear envelope via the SUN–KASH protein complex [2].

Meanwhile, a question arises whether there are any interactions between nonhomologous chromosomes during meiosis when the cell nucleus, nuclear envelope, and chromosomes undergo many changes. In somatic cells, chromosomes occupy a certain space within the nucleus, termed chromosome territories [3,4]. Although chromosome territories may differ among tissues of the same organism [5], their locations were evolutionarily conserved in primates and humans [6,7]. Functionally, the three-dimensional genome organization regulates gene expression via crosstalk between DNA elements located in specific chromosomal territories. Regulatory nucleus organization may involve intra- and interchromosomal interactions, with genomic activity being situated in specific hubs [8]. The points of overlap or intermingling of chromosomal territories are metaphorically called ‘kissing chromosomes‘ and, in general, this phenomenon has been termed nonhomologous chromosomal contacts (NHCCs) [9]. Such nonhomologous chromosome interactions are also characteristic of germ cells. During prophase I, meiotic chromosomes keep their compartment structure, but topologically associated domains disappear [10,11]. The contacts of the asynaptic axes of nonhomologous chromosomes (nonhomologous synapsis) can occur in hybrid organisms (for example, between autosomes or between sex chromosomes and autosomes) [12,13,14,15].

NHCCs can be mediated by centromeric and telomeric regions. The phenomenon of ‘nonhomologous centromere coupling’ or centromere clustering is known to occur in budding yeast and drosophila, in which pairing between centromeres of nonhomologous chromosomes precedes homology-dependent synapsis [16,17,18,19]. Contacts between stretched centromeres of nonhomologous chromosomes have been observed in interspecific mole vole hybrids [20]. Interchromosomal contacts via centromeric heterochromatin and chromocenters are possible [21,22,23]. In nematodes, telomeric ends of chromosomes are grouped through SUN1 patches until homologs are recognized [24]. Telomeric clustering as a variant of the non-homologous chromosome interaction is also present in other organisms [25,26].

The rarest case is a nonhomologous chromosome contact via the SYCP3 (synaptonemal complex protein 3) filament. This type of interaction was first described for irradiated mice [27]. We first described the natural phenomenon of nonhomologous chromosome contacts in prophase I of meiosis in *Ellobius alaicus*, an unusual rodent species, which has two isomorphic X chromosomes in both sexes [20]. Such contacts resemble the specialized chromosomal regions in which the short arms of nonhomologous chromosomes extend, contact (‘touching’ type), partly fuse with each other (‘anchoring/tethering’ type), and even follow a pattern of a complete fusion (‘fusion’ type). These findings, and the fact that the Alay mole vole is a species with Robertsonian variability [28,29], allowed us to assume that such meiotic contacts may be a prelude to a Robertsonian translocation and could have taken place in the evolutionary history of this species.

During gametogenesis, the nucleus undergoes drastic changes. They are complicated by inconsistencies in the functional movements of chromosomes and their stable position due to specific chromosomal territories. Such processes as meiotic silencing and chromatin reorganization, SC formation, and chromosome–nuclear envelope interaction in prophase I are still poorly understood and offer a promising field for exploration [30,31]. Our previous paper [20] dealt with the various types of nonhomologous chromosome contacts at the pachytene stage. An in-depth analysis of this phenomenon seems to be relevant. We are particularly interested in the study of meiotic nonhomologous contacts in relation to DNA DSBs formation, as well as their heterochromatic environment during prophase I. One of the tasks was to answer the question: what types of contacts are present in the zygotene and diplotene. Thus, here we aimed to understand the genesis of nonhomologous interactions and to thoroughly characterize meiotic contacts with a focus on their dynamics during prophase I.

## 2. Materials and Methods

Chromosomal samples of three Alay mole voles *E. alaicus*, originating from south-western Kyrgyzstan, were received from the cytogenetic collection (a part of the joint collection of wildlife tissues for fundamental, applied and environmental research of the Koltzov Institute of Developmental Biology RAS, Core Centrum of the Koltzov Institute of Developmental Biology RAS, state registration number 6868145). All manipulations with animals were carried out according to the international rules and the rules of the Ethic*s* Committee of Vavilov Institute of General Genetics RAS (order No. 3 of 10 November 2016) and the Ethics Committee for Animal Research of the Koltzov Institute of Developmental Biology RAS (the most recent protocol is numbered 37—25 June 2020).

Synaptonemal complex preparations were made and fixed according to Peters et al. [32], with some modifications [33]. Primary antibodies used for immunostaining: rabbit anti-synaptonemal complex protein 3 (SYCP3) antibodies (diluted 1:250, Abcam, Cambridge, UK); human anti-centromere Calcinosis Raynaud’s phenomenon, Esophageal dysmotility, Sclerodactyly, and Telangiectasia (CREST) antibody (CREST, 1:250, Fitzgerald Industries International, Acton, MA, USA); mouse anti-phospho-histone H2AX, also known as γH2AFX (diluted 1:250–500, Abcam), rabbit anti-H3K9me3 antibody (1:100, Abcam). As secondary antibodies, we used goat anti-rabbit IgG, Alexa Fluor 488-conjugate (Invitrogen, Carlsbad, CA, USA); goat anti-human IgG, Alexa Fluor 546-conjugate (Invitrogen, Carlsbad, CA, USA); goat anti-mouse IgG, Alexa Fluor 546-conjugate; and IgG, Alexa Fluor 555-conjugate (Invitrogen, Carlsbad, CA, USA) (diluted 1:300–800). Slides were washed in phosphate-buffered saline (PBS) and immersed into Vectashield with 4′,6-diamidino-2-phenylindole (DAPI) (Vector Laboratories, Burlingame, CA, USA). Slides were analyzed using a fluorescence light microscope Axio Imager D1 (Carl Zeiss, Jena, Germany). Immunostaining procedure was described previously [34,35].

Inter-centromeric distances of meiotic contacted acrocentric chromosomes were measured using the MicroMeasure program (Colorado State University, Fort Collins, CO, USA). The statistical analysis of all data was performed using GraphPad Prism 9 software (San Diego, CA, USA). Mean values (M) and standard deviation (SD) were calculated by the descriptive option of the software. P-values reported in Table S1 were calculated by Mann–Whitney two-sided non-parametric test by the special option of the software.

## 3. Results

All studied *E. alaicus* specimens had karyotypes with 2n = 52 and NF = 56, as the most previously analyzed Alay mole voles [20,29].

The stepwise analysis of meiotic prophase I made it possible to evaluate temporal and spatial interactions of homologous and nonhomologous chromosomes. To this end, we studied: (i) the localization of the SYCP3 protein, the main component of axial elements (AEs), and lateral elements (LEs) of the SC; (ii) kinetochore proteins (by means of an anti-CREST antibody); (iii) the γH2AFX protein, a marker of DSBs, asynaptic chromosomal regions, and the sex body; and (iv) H3K9me3, as a hallmark of heterochromatin. To clearly identify the meiotic prophase I stages, we use the criteria described in several articles [36,37,38,39]. In the mid-late zygotene, γH2AFX positive asynaptic sites are present, and an intense γH2AX signal is located around the XX bivalent that can occupy the entire space of the nucleus. Axial elements and bivalents seem to be slightly stretched or under slight tension. In the early–mid diplotene, the XX bivalent is shrouded in a weak γH2AFX signal, desynaptic sites do not have γH2AFX foci. Closer to the middle of the diplotene, the bivalents have an alternation of brighter and less bright SYCP3 regions.

**Figure 1:**
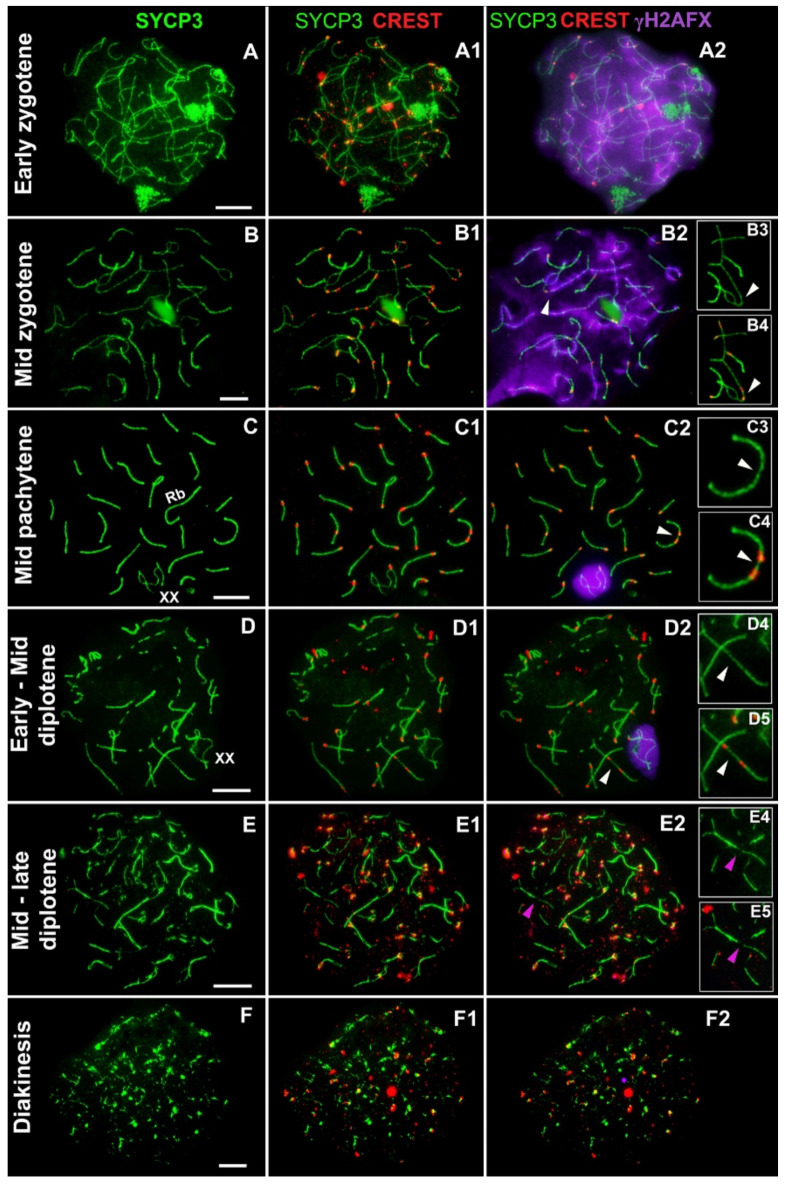
Meiotic prophase I in studied *E. alaicus* specimens. Axial and lateral elements of the SC were identified using an anti-SYCP3 antibody (green), kinetochores were detected by an anti-CREST antibody (red), and chromatin inactivation was revealed using an anti-γH2AFX antibody (violet). In early zygotene, axial elements emerge and begin to enter synapsis (**A**–**A2**). The entire meiotic nucleus space is filled with the γH2AFX signal (**A2**). In mid-zygotene, when almost all homologs begin to synapse, usually (but not always) asynaptic zones of chromosomes are wrapped in a cloud of γH2AFX (**B**–**B2**). The space between chromosomes contains no γH2AFX signal (**B2**). At this stage, contacts of nonhomologous chromosomes’ pericentromeric sites are noticeable: one axial element is in contact with the bivalent (arrow, **B3**,**B4**). At the mid pachytene stage, all bivalents complete synapsis, and the sex bivalent is displaced to the periphery of the nucleus and capped by a γH2AFX cloud (**C**–**C2**). Meiotic contact of nonhomologous chromosomes is present in this cell: the two bivalents are connected through the SYCP3 bridge that has formed between the two centromeric regions (arrow, **C2**–**C4**). In diplotene, SC fragmentation (**D**–**D2**) and homolog desynapsis (**E**–**E2**) are observed. Meiotic contacts are still visible in early–mid diplotene (**D4**,**D5**), whereas in late diplotene, SYCP3 bridges and SYCP3 contacts are not detectable (magenta arrowhead) because the SYCP3 protein is eliminated from the axis (**E**–**E2**). In diakinesis, homologs desynapse and SYCP3, as a clump, is typically visible around the centromeres (**F**–**F2**). Scale bar = 5 µm.

It was found that in early zygotene, axial elements arise, and some of them begin to enter synapsis (Figure 1(A,A1)). The entire nucleus is filled with the γH2AFX signal (Figure 1(A2)). In mid zygotene, the axes become shorter, most of them enter the synapsis and usually start to synapse from terminal regions (Figure 1(B,B1)). Most of the asynaptic chromosome segments proved to be surrounded by the γH2AFX signal (Figure 1B). Some bivalents contact each other; for example, as demonstrated in Figure 1(B2–B4), the centromeric region of one of the bivalents (with a short asynapsis region in the center) is in contact with the centromeric region of the axial element of the other bivalent. In mid pachytene, all bivalents complete synapsis, whereas the sex bivalent is localized to the peripheral part of the nucleus and is enveloped by γH2AFX (Figure 1(C,C1,C2)), as previously described for closely related species *E. talpinus* and *E. tancrei* [34,36]. Contacts between some bivalents are kept at this stage; Figure 1(C2–C4) demonstrate how two bivalents contact each other in centromeric regions with the formation of a SYCP3 filament between the short arms. Starting from the late zygotene there were no γH2AFX signals in the regions of meiotic contacts. In diplotene, desynapsis proceeds via fragmentation of SYCP3 axes (Figure 1(D,D1,E,E1)), and there is a decrease in the γH2AFX signal within the sex bivalent (Figure 1(D2)) down to its absence (Figure 1(E2)). In early–mid diplotene, meiotic contacts of bivalents are visible (Figure 1(D4,D5)), whereas in late diplotene, the SYCP3 protein is sequentially eliminated from the axes, and thus interbivalent connections are not detectable by immunocytochemical analysis (Figure 1(E1,E2)). In diakinesis, the homologs desynapse and SYCP3 signals in the form of clumps and lumps, as a rule, are seen in the centromere regions (Figure 1(F,F1,F2)).

**Figure 2:**
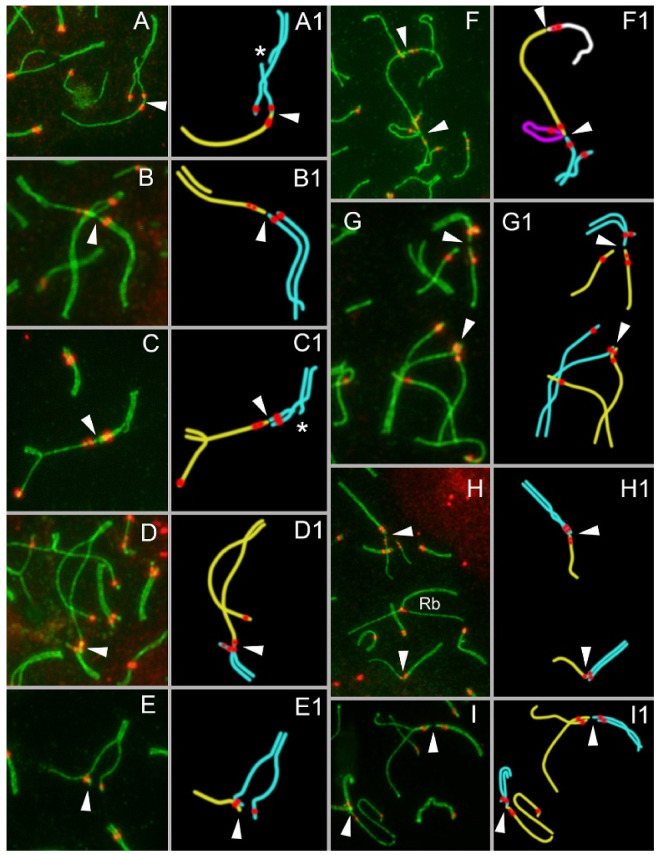
Zygotene configurations of nonhomologous chromosome contacts during meiosis in studied *E. alaicus* specimens. Axial and lateral elements of the SCs were identified using the anti-SYCP3 antibody (green). Kinetochores were detected by means of the anti-CREST antibody (red). The arrow indicates the contact of nonhomologous chromosomes. The contacting chromosomes in zygotene are at different stages of synapsis (asynchronous synapsis) (**A**–**I**). The chromosomes, in which synapsis begins and ends earlier than that in the nonhomologous partner, are highlighted in cyan. Chromosomes with delayed synapsis (including univalents) are shown in yellow, white, or magenta. The contacting axes sometimes contain a break marked by an asterisk (**A1**,**C1**).

**Figure 3:**
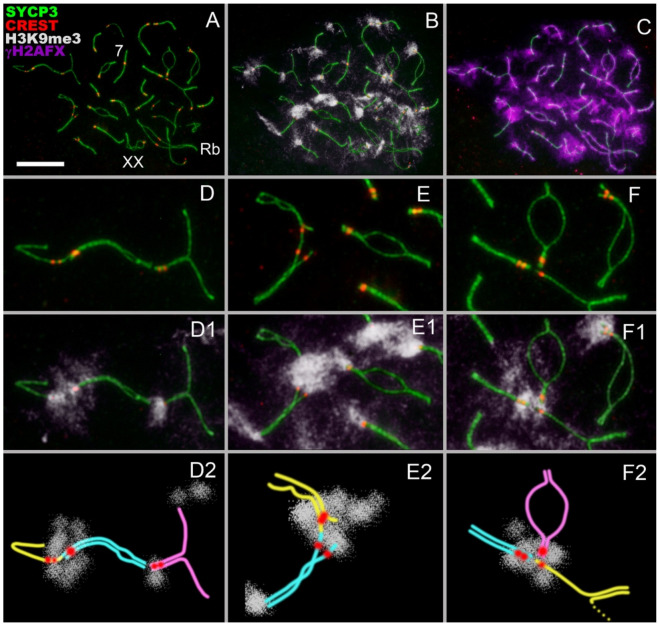
Meiotic contacts of nonhomologous chromosomes in zygotene and H3K9me3 histone distributions in *E. alaicus* spermatocytes. Immunostained nucleus (**A**–**C**), its enlarged parts (**D**–**F**,**D1**–**F1**), and schemes of meiotic contacts (**D2**–**F2**) are presented. Axial and lateral elements of the SCs were visualized using the anti-SYCP3 antibody (green). Kinetochores were detected using the anti-CREST antibody (red). Heterochromatin was detected by means of an anti-H3K9me3 antibody (white). Chromatin inactivation was revealed using an anti-γH2AFX antibody (violet). The non-Robertsonian chromosome is designated as 7 according to the karyotype [28,29]. The contact regions of nonhomologous bivalents are localized in the H3K9me3 clouds. Scale bar (A–С) = 5 µm.

**Figure 4:**
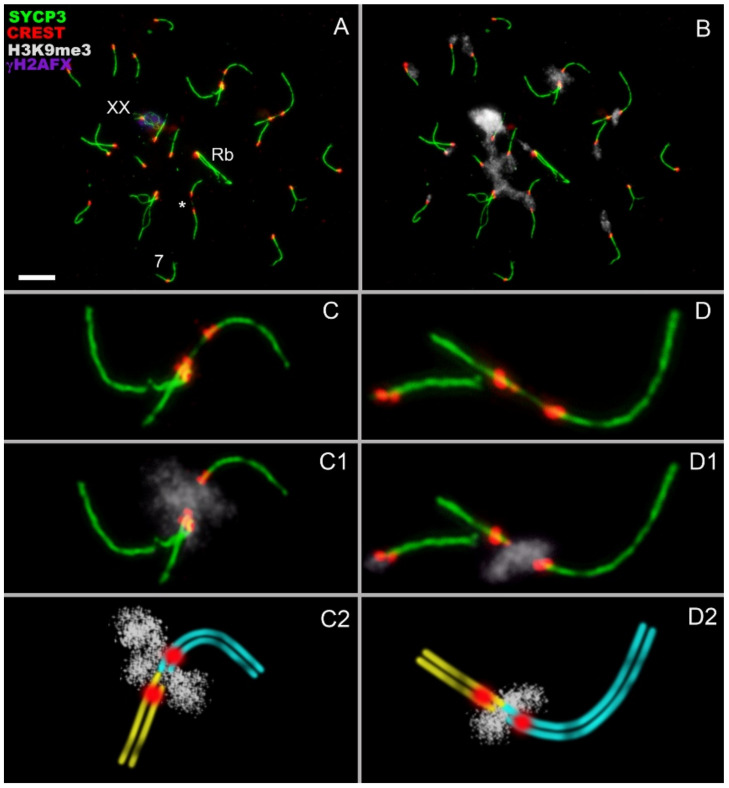
Meiotic contacts of nonhomologous chromosomes and H3K9me3 histone distributions in diplotene of *E. alaicus* spermatocytes. Immunostained nuclei (**A–D**,**C1**,**D1**) and schemes of meiotic contacts (**C2**,**D2**) are presented. Lateral elements of the SCs were visualized using the anti-SYCP3 antibody (green). Kinetochores were detected using the anti-CREST antibody (red). Heterochromatin was detected by means of an anti-H3K9me3 antibody (white). Chromatin inactivation was revealed using an anti-γH2AFX antibody (violet). XX bivalent has a weak γH2AFX signal (**A**). The non-Robertsonian chromosome is designated as 7 according to the karyotype (A). The contact regions of nonhomologous bivalents are localized in the H3K9me3 clouds. The proximity type of contact marked by an asterisk. Scale bar (A,B) = 5 µm.

**Figure 5:**
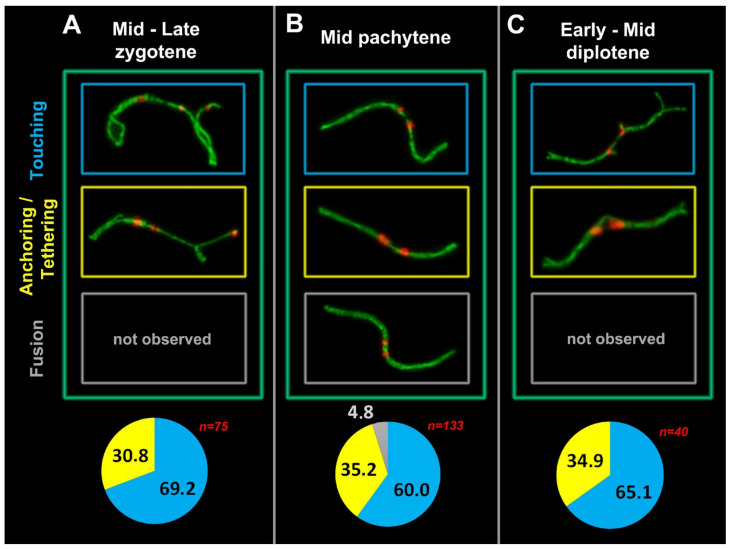
Types of meiotic contacts at the different prophase I substages in *E. alaicus*: mid–late zygotene (**A**), mid pachytene (**B**) and early–mid diplotene (**C**). ‘Touching’, ‘anchoring/tethering’, and ‘fusion‘ types were counted in cells with chromosome connections only. Proportions of connection types (bottom diagrams): blue color corresponds to ‘touching’, yellow to ‘anchoring/tethering’, and gray to ‘fusion’. ‘Fusion’ type was found only at the pachytene. Red numbers are the counted spermatocytes. The color of the frames corresponds to the types of contacts. Microphotos and data from B section were taken from [20].

Mid–late zygotene (Figure 2 and Figure 3), mid pachytene and early–mid diplotene (Figure 4) were studied in more detail (Figure 5) and are shown in Table S1 for all stages. In the analyzed stages, the number of meiotic contacts per nucleus varied from 1 to 4, with an average of 1.05 for the zygotene, 1.3 for the pachytene, and 1.13 for the diplotene, as shown in Table S1, if nuclei with chromosomal connections are considered. ‘Touching’ and ‘anchoring/tethering’ types of contacts have been identified in zygotene, pachytene, and diplotene. The ‘fusion’ type has only been found in pachytene (Figure 5). To assess the dynamics of non-homologous chromosomal interactions, a morphological characterization (structure and behavior) of the axial and lateral elements and a morphometric analysis (inter-centromeric distance) of the contact regions of two SC bivalents were carried out.

From zygotene to diplotene, the distance between the centromeres of two contacting bivalents varied from 0.11 µm to 0.14 µm for touching type, from 0.09 µm to 0.11 µm for ‘anchoring/tethering‘ type, and it was 0.10 µm for fusion type (Table S1). The inter-centromere distances are significantly different in zygotene and diplotene for ‘touching’ and ‘anchoring/tethering‘ types of contacts. Additionally, what is most interesting, the distances between centromeres differ significantly in zygotene and pachytene for ‘anchoring’ contacts.

Examination of meiotic nonhomologous contacts of zygotene bivalents revealed several features:

1. The contact of axial elements occurs before the complete synapsis of homologs (Figure 2A–I and Figure 3A–F);
2. Asynchronous synapsis of the chromosomes involved in contact takes place: synapsis of one of the bivalents is faster than that of the other (Figure 2A,I);
3. Occasionally, a gap in the axial/lateral element is seen in the bivalent that manifests faster synapsis (Figure 2(A,A1,C,C1)). This feature was detected in 8 out of 75 nuclei;
4. Typically, the contact region is surrounded by the γH2AFX signal until synapsis is complete (Figure 1(B2)). In pachytene and diplotene, there is no γH2AFX signal in the contact zone (Figure 1(C2) and Figure 4A);
5. In zygotene, various chromosomes enter into meiotic contacts, as was demonstrated earlier for pachytene bivalents [20].

Zygotene chromosomal connections correspond to different types of pachytene contacts: (1) ‘proximity‘ (Figure 2(G,G1), top), (2) ‘touching‘ (Figure 2(B,B1,E,E1,G,G1,H,H1,I,I1,top)), and (3) ‘anchoring/tethering‘ (Figure 2(A,A1,C,C1,D,D1,F,F1,H,H1,I,I1,bottom,D,E), Table S1). If the centromeres had spatial proximity and the SYCP3 filament in the inter-centromere space was discontinuous (with gap or gaps), then such contact was considered as a ‘touching’ (Figure 2(E,G, bottom)). Diplotene chromosomal interactions correspond to types of zygotene and pachytene contacts: (1) ‘proximity‘ (Figure 4A), (2) ‘touching‘ (Figure 5), and (3) ‘anchoring/tethering‘ (Figure 4(C,C1,C2,C), Table S1). In diplotene XX, bivalent has a weak γH2AFX signal (Figure 4A).

We determined H3K9me3 (histone 3 lysine 9 trimethylation) immunolocalization in zygotene (Figure 3), pachytene (Figure S1) and diplotene (Figure 4) spermatocytes. H3K9me3 is an epigenetic modification, which is known to be involved in the regulation of a broad range of biological processes, including the formation of transcriptionally silent chromatin, a heterochromatin [40]. Most zygotene bivalents had H3K9me3-positive pericentromeric regions (Figure 3B). Different types of contacts of zygotene bivalents turned out to be located within H3K9me3 clouds (Figure 3(D–D2,E–E2,F–F2)), similarly to our previous findings at the pachytene stage [20]. Most of the diplotene bivalents had H3K9me3 clouds, but they were smaller and less bright (Figure 4). Despite the fact that the vast majority of meiotic contacts had a lot of H3K9me3 signals, some of these connections were H3K9me3-poor or had no H3K9me3 foci at all, as shown in Figure S1.

## 4. Discussion

The organization of the nucleus and chromosome interactions within it have been actively investigated since the late 19th century and early 20th century [41,42] to the present day [23,43,44]. Inter-chromosomal nonhomologous contacts are generally restricted by the protective function of telomeres [45,46], although such interactions are normally possible if chromosome integrity is not compromised [16,17,21,22,23]. Our previous studies have identified a new type of a nonhomologous chromosome interaction during prophase I of meiosis, namely, meiotic bivalent contacts through the modification of SYCP3-positive axial/lateral elements of the short arms of acrocentrics [20]. We have described different types of meiotic contacts in pachytene: (1) ‘proximity´, (2) ‘touching,’ (3) ‘anchoring/tethering’, and (4) ‘fusion’. Research on nonhomologous interactions in early meiosis is particularly interesting because the key events of prophase I occur here. Chromosome morphogenesis and chromatin changes during these stages include: (1) interhomolog interactions, including homology recognition, meiotic recombination, and SC formation [1,47]; (2) the assembly of a chromosome bouquet, which is believed to facilitate the search for homologs by bringing ends of chromosomes closer and aligning them through coordinated movements near the nuclear envelope [2,48,49]; (3) a complex program of gene expression, which unfolds when a low level of transcription is manifested in leptotene and zygotene, as well as when transcription is reactivated in pachytene [39]; (4) epigenetic reorganization of chromatin, e.g., its phosphorylation, ubiquitination, methylation, sumoylation, inducing a silent state (reviewed by [50]).

In the present paper, we concentrated on the meiotic contacts from leptotene to diplotene. We can assume that meiotic contacts of bivalents start as early as in leptotene, but it is difficult to detect them because the main immunomarker, the SYCP3 protein, is only beginning to build axial elements at this point and cannot give a picture of a chromosome interaction. Similarly, the elimination of this protein from diplotene bivalents does not allow us to assess the stage and degree to which these contacts are retained.

The number of contacts in the mid pachytene is significantly greater than that in the zygotene and diplotene, and the distance between centromeres is also the smallest for all types of contacts (Figure 5), as shown in Table S1. The duration of the meiotic stages is different; moreover, the speed of movement of chromosome ends, which are tethered to the nuclear envelope, varies. In mice, telomere movements were rather slow at the bouquet stage, faster in zygotene, and significantly decreased during pachytene [51]. All the movements were correlated with telomere distribution; therefore, our data on the specificity of inter centromeric distances are in accordance with longevity of the stages and dynamics of chromosome movements.

It is noteworthy that just the first three types of contacts are identifiable in zygotene. The ‘fusion’ type is detectable exclusively in pachytene and only in 5% of all meiotic contacts [20]. This makes logical sense because the synapsis of contacting bivalents ends in the early-to-mid pachytene, and only then are we able to immunodetect a fusion or a very close contact of lateral SC elements near short arms of two bivalents.

We checked the localization of the repressive chromatin marker, H3K9me3, in the contact region between two bivalents. H3K9me3 is associated with constitutive centromeric heterochromatin [40,52] and chromocenters [23]. Heterochromatin involvement in nonhomologous centromere associations in chromocenters [23] may explain the SYCP3-positive inter-bivalent contacts that we identified within H3K9me3 clouds in mole vole spermatocytes. It is likely that the nonhomologous interaction of acrocentrics starts in the leptotene or zygotene stages (or earlier), precisely within the H3K9me3-positive chromocenters. However, it is still difficult to explain the presence of contacts within the H3K9me3 clouds between some bivalents and their absence between others.

It should be pointed out that the bivalent contacts did not introduce any alterations into the classic pattern of DSBs distribution. It is likely that the regions of meiotic contacts in the zygotene are not specific for the functioning of DSB repair machinery but simply fall under the general processes occurring in chromatin. Double-strand breaks are repaired, the number of asynaptic sites decreases, and the γH2AFX foci consistently disappears in the contact region. The presence of contacts does not lead to any violations of homologous synapsis and meiotic progression up to diplotene stage. The result may explain the low effect of negative selection against Rb translocation. Since selection does not eliminate the rearranged chromosomes in meiosis, they have a chance to pass into gametes and survive in natural populations of *E. alaicus* [28,29].

This paper is also intended to determine important points for future research. In this work, we were unable to answer a number of questions. How do the axial elements fuse with each other? This is an important issue because the chromosome ends are connected to the nuclear envelope through the interaction of the shelterin complex and the SUN–KASH system. Do the axial elements detach from the nuclear envelope or is this due to protein-binding complexes during attachment? Do meiotic contacts persist after diplotene when the SYCP3 protein is eliminated? Additionally, perhaps the most important question, what is the biological meaning of these meiotic contacts? These questions will be addressed in future studies.

## Data Availability

Not applicable.

## Supplementary Materials

The following supporting information can be downloaded at: https://www.mdpi.com/article/10.3390/genes13122196/s1, Table S1: Data on meiotic non-homologous contacts in *Ellobius alaicus*. Figure S1: Meiotic contacts of nonhomologous chromosomes in pachytene and H3K9me3 histone distributions in *E. alaicus* spermatocytes.
