# Supplementary material for: Nonhomologous Chromosome Interactions in Prophase I: Dynamics of Bizarre Meiotic Contacts in the Alay Mole Vole Ellobius alaicus (Mammalia, Rodentia)
Source: Genes (Basel). 2022 Nov 23;13(12):2196. doi: 10.3390/genes13122196 (PMC9778597; doi:10.3390/genes13122196)
Supplement: Supplementary file 1 [file genes-13-02196-s001.zip › genes-1971257-supplementary.pdf]

Supplementary materials

Nonhomologous Chromosome Interactions in Prophase I: Dynamics of Bizarre Meiotic Contacts in the Alay Mole Vole *Ellobius alaicus* (Mammalia, Rodentia)

Sergey Matveevsky [1, *, †], Irina Bakloushinskaya [2, †], Valentina Tambovtseva [2], Maret Atsaeva [1, 3], Tatiana Grishaeva [1], Aleksey Bogdanov [2], Oxana Kolomiets [1]

[1] Vavilov Institute of General Genetics, Russian Academy of Sciences, 119991 Moscow, Russia;

[2] Koltzov Institute of Developmental Biology, Russian Academy of Sciences, 119334 Moscow, Russia

[3] Chechen State University, 364024 Grozny, Chechen Republic, Russia

* Correspondence: sergey8585@mail.ru (S.M.)

† These authors contributed equally to this work

==

Table S1. Data on meiotic non-homologous contacts in *Ellobius alaicus*

		1	2	3	4	5	6
	**Meiotic prophase I substages**	**Total number of nuclei**	**Total number of nuclei with meiotic contacts**	**Inter-centromeric distance in meiotic contacts, µm**			**Average number of contacts per nucleus, M±SD, (*n* nuclei), [range]**
				**"Touching" type, M±SD (*n* nuclei)**	**"Anchoring / Tethering" type, M±SD, (*n* nuclei)**	**"Fusion" type, M±SD, (*n* nuclei)**	
**a**	**Mid - late zygotene**	150	75	**0.13**±0.03, (*n*=22)	**0.09**±0.03, (*n*=25)	–	**1.05**±0.2, (*n*=75), [1-2]
**b**	**Mid pachytene***	160	66	**0.11**±0.03, (*n*=22)	**0.11**±0.02, (*n*=24)	**0.10**±0.02, (*n*=9)	**1.30**±0.6, (*n*=66), [1-4]
**c**	**Early - mid diplotene**	87	40	**0.14**±0.04, (*n*=14)	**0.10**±0.04, (*n*=15)	–	**1.13**±0.4, (*n*=40), [1-3]
	**Significant (P < 0.05)**			3a/4a, 4a/4b, 3c/4c			6a/6b
	**Not significant**			3a/3b, 3a/3c, 4a/4d, 3b/4b, 3b/5b, 4b/5b,3b/3c, 4b/4c			6a/6c, 6b6c
	* part of the data is taken from Matveevsky et al., 2020. Data on the proportion of contact types (Figure 5 in article) are taken from Matveevsky et al., 2020						

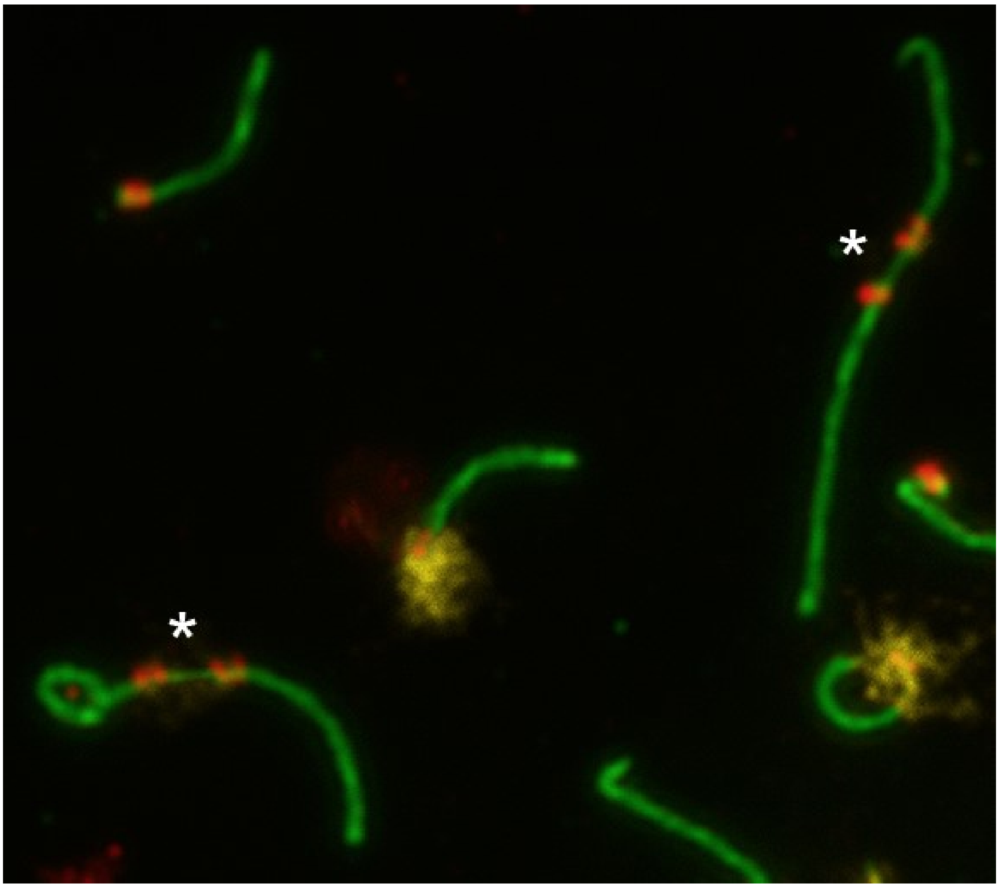

Figure S1. Meiotic contacts of nonhomologous chromosomes in pachytene and H3K9me3 histone distributions in *E. alaicus* spermatocytes. Lateral elements of the SCs were visualized using the anti-SYCP3 antibody (green). Kinetochores were detected using the anti-CREST antibody (red). Heterochromatin was detected by means of an anti-H3K9me3 antibody (yellow). Regions of chromosome contacts were marked by an asterisk.
